# Bidirectional two-sample Mendelian randomization analysis investigates causal associations between cathepsins and inflammatory bowel disease

**DOI:** 10.3389/fgene.2024.1436407

**Published:** 2024-09-18

**Authors:** Na Wang, Jun Liu, Bao Chai, Jianhong Yao, Xufang Du, Qi Mei, Xuena Wang

**Affiliations:** ^1^ Shanxi Bethune Hospital, Shanxi Academy of Medical Sciences, Tongji Shanxi Hospital, Third Hospital of Shanxi Medical University, Taiyuan, China; ^2^ Department of Oncology, Tongji Hospital, Tongji Medical College, Huazhong University of Science and Technology, Wuhan, Hubei, China

**Keywords:** IBD, cathepsins, mendelian randomization, genetics, genome-wide association studies

## Abstract

**Background:**

Cathepsins, key regulators of the pathology of gastrointestinal disorders such as inflammatory bowel disease (IBD), are a target protease that has attracted much attention in recent years. IBD is a chronic and relapsing inflammatory disorder of the gut. Traditional studies have shown a correlation between cathepsin and the risk of IBD, while the causal relationship remains unclear.

**Methods:**

This study utilized Mendelian randomization techniques to evaluate the causal relationships between eleven cathepsins and the subtypes of IBD, such as ulcerative colitis (UC) and Crohn’s disease (CD). We also performed a series of sensitivity analyses to validate the primary Mendelian randomization (MR) results, including Cochran’s Q test, the MR-PRESSO global test, and the MR pleiotropy test.

**Results:**

The forward MR analyses showed no significant association between cathepsins and IBD. Reverse Mendelian randomization analyses suggested that UC might lead to elevated cathepsin G levels [inverse-variance weighted (IVW): *p* = 0.038, b = 9.966], and CD might cause a decrease in cathepsin B levels [IVW: *p* = 0.002, b = −10.525] and cathepsin L1 levels [IVW: *p* = 0.045, b = −4.742].

**Conclusions:**

Our findings offer novel and comprehensive evidence on the impact of UC or CD on cathepsins, potentially providing valuable insights into the treatment and prognosis of IBD.

## 1 Introduction

Inflammatory bowel disease (IBD) is a chronic inflammatory condition affecting the intestine, mainly defined as either ulcerative colitis (UC) or Crohn’s disease (CD) (Guan, 2019; Rogler et al., 2021; Yang et al., 2021; Nóbrega et al., 2018; GBD, 2017 Inflammatory Bowel Disease Collaborators, 2020). In recent years, IBD has emerged as a significant global public health problem due to its elevated rates of morbidity and mortality, presenting a substantial threat to human health and well-being (Mak et al., 2020; Hasan et al., 2020; Nielsen et al., 2022). Previous reports indicate that long-term IBD patients are at increased risk of gastrointestinal and extraintestinal malignancies. For instance, individuals with UC exhibit a heightened risk of developing pancreatic cancer (PC), with an odds ratio (OR) of 1.18 and a 95% confidence interval (CI) of 1.07–1.31^9^ (Huang et al., 2023). A Mendelian randomization study reported that IBD may be a risk factor in the development of oral cavity cancer (IBD: OR = 1.180, 95% CI: 1.059 to 1.316, *p* = 0.003; CD: OR = 1.112, 95% CI: 1.008 to 1.227, *p* = 0.034; UC: OR = 1.158, 95% CI: 1.041 to 1.288, *p* = 0.007) and breast cancer (IBD:OR = 1.059; 95% CI: 1.033 to 1.086; *p* < 0.0001; CD:OR = 1.029; 95% CI: 1.002 to 1.055; *p* = 0.032). Other studies have demonstrated a 17% greater overall cancer risk among IBD patients than those without the condition (Derikx et al., 2017; Singh et al., 2014; Faye et al., 2022).

Cathepsins are frequently used to catalyze the hydrolysis of unwanted proteins, play a key role in disease progression, and are important mediators of many inflammatory diseases (Reiser et al., 2010). Dysregulated cathepsins have been implicated in numerous pathologies, including arthritis, pancreatitis, and atherosclerosis (Yadati et al., 2020; Patel et al., 2018). Specific cathepsins have been identified as serological markers for certain diseases (Kyriakidi et al., 2016). In recent years, the role of cathepsins (such as cathepsin B, D, G, and L) has been widely studied in IBD (Menzel et al., 2006; Dong et al., 2022; Zhao et al., 2018). Studies reveal a significant increase in the cathepsin D protein expression in inflamed intestinal mucosa from IBD patients compared to non-inflamed mucosa (Hausmann et al., 2004). Inhibition of cathepsin D was followed by amelioration of mouse dextran sodium sulfate (DSS) colitis (Menzel et al., 2006); the level of anti-cathepsin G antibodies was significantly higher in patients with severe colitis than in those with mild or moderate colitis (*p* < 0.05) (Kuwana et al., 2000). Notably, Marta Dabek’s study also verifies this point that cathepsin G has been found to be overexpressed in patients with UC compared to healthy controls (Dabek et al., 2009). Similar results have been found with other cathepsins, such as Zhao et al. (2018) and Zhao et al. (2016). However, our understanding of the association between other members of the cathepsin family and IBD remains limited, warranting further exploration and investigation. Meanwhile, due to the methodological constraints of observational studies, causal relationships between cathepsins and IBD cannot be conclusively established.

Genetics play a role in predisposition to IBD. The genetic risk ratio for IBD is in the range of 15–42 for CD (Monsén et al., 1991; Probert et al., 1993; Küster et al., 1989; Meucci et al., 1992; Orholm et al., 1991) and 7–17 for UC (Meucci et al., 1992; Orholm et al., 1991). Heredity as an etiological factor is stronger in Crohn’s disease than in UC. Mendelian randomization (MR) analysis supplies a promising approach to exploring potential causal associations by leveraging instrumental variables (IVs) that are independent of common confounders, thus overcoming the limitations of traditional observational studies to some extent (Emdin et al., 2017; Smith and Ebrahim, 2004; Sekula et al., 2016). Recently, MR analysis has emerged as a tool for investigating relationships between IBD and various factors with the expanding size and scope of genome-wide association studies (GWAS) (Freuer et al., 2022; Zeng et al., 2023; Lund-Nielsen et al., 2018; Jones et al., 2020; Saadh et al., 2023). Long-term IBD patients have been reported to face an up to three- to five-fold increased risk of developing colitis-associated colorectal cancer. To determine whether a potential causal relationship exists between cathepsins and IBD, we conducted a bidirectional two-sample MR analysis utilizing publicly available GWAS datasets.

## 2 Materials and methods

### 2.1 Study design

**FIGURE 1:**
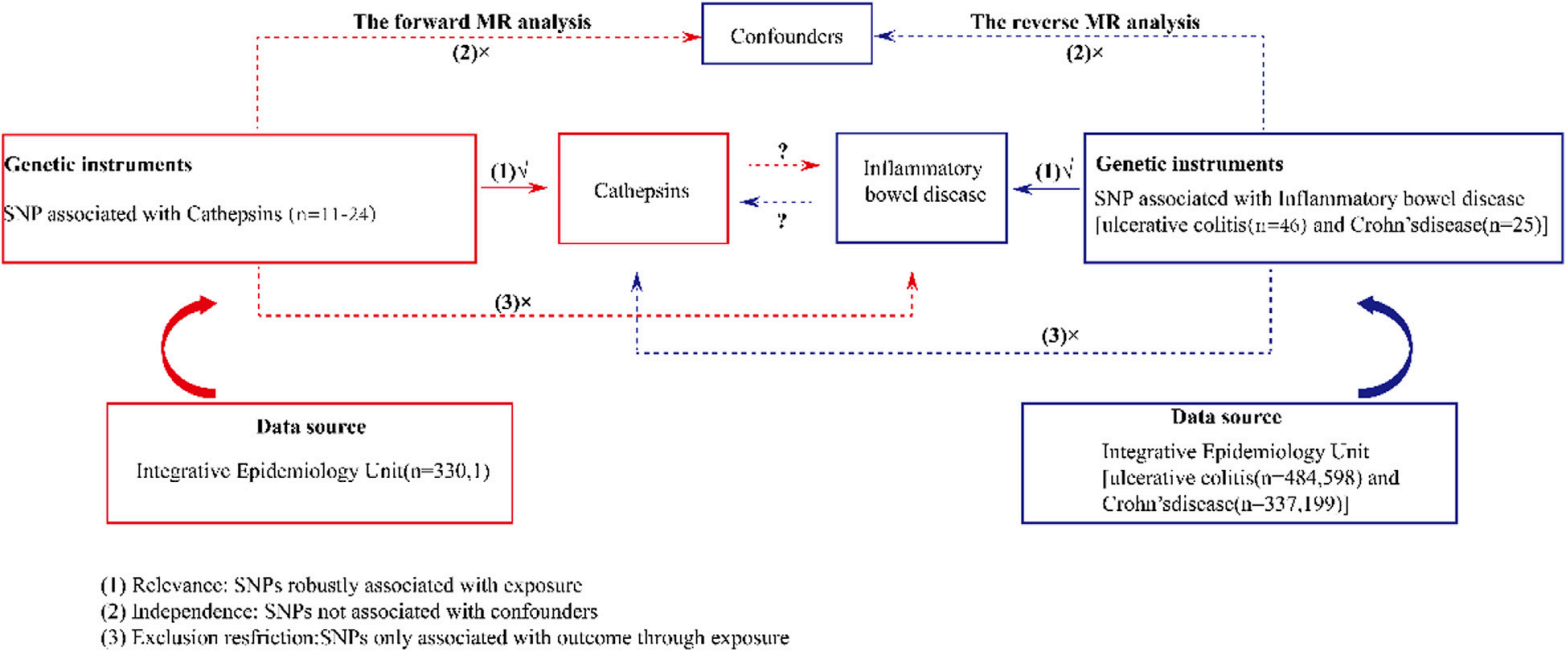
Schematic design of a bidirectional two-sample Mendelian randomization study of cathepsins and IBD. This design assumes a bidirectional association between cathepsins and IBD. Genetic variants can influence IBD through cathepsins and *vice versa*. SNP, single-nucleotide polymorphism.

MR was employed to investigate the relationships between various cathepsins and IBD. The design of our study is shown in Figure 1. Initially, we extracted genetic variants that serve as IVs for 11 cathepsins. Subsequently, we collected the summary data, including all single-nucleotide polymorphisms (SNPs) derived from GWASs of UC and CD. Next, we performed bidirectional MR analyses with five distinct MR methods, including MR-Egger, weighted median, inverse-variance weighted (IVW), simple mode, and weighted mode. Furthermore, we conducted a series of sensitivity analyses, comprising Cochran’s Q test, the MR-PRESSO global test, and the MR pleiotropy test, to evaluate the heterogeneity and horizontal pleiotropy of the MR results. Because the data employed in this study were based on published studies and public databases, no additional ethical approval from an institutional review board was necessary.

### 2.2 Data sources

The summary-level GWAS data of 11 cathepsins (µg/L) (including cathepsin B, D, E, F, G, H, L1, L2, O, S, and Z) and IBD (including UC and CD) were sourced from the Integrative Epidemiology Unit (IEU) open GWAS project (https://gwas.mrcieu.ac.uk/). This project, supported by the MRC IEU at the University of Bristol, collated and analyzed GWAS data from different sources, such as the UK Biobank, published articles, and the FinnGen Biobank. The approval of the Ethical Review Authority was not required because the data used in our study were public, anonymized, and de-identified. Further details regarding the exposure and outcome datasets are supplied in Table 1.

**TABLE 1:** Information about the exposures and outcome datasets.

IEU GWAS id	Exposure or outcome	Identified SNPs	Participants included in the analysis	F-statistic
prot-a-718	Cathepsin B	19	3,301 individuals of European descent	34.297
ebi-a-GCST90012053	Cathepsin D	20	21,758 individuals of European descent	92.98
prot-a-720	Cathepsin E	11	3,301 individuals of European descent	21.902
prot-a-722	Cathepsin F	13	3,301 individuals of European descent	35.678
prot-a-723	Cathepsin G	15	3,301 individuals of European descent	22.807
prot-a-725	Cathepsin H	11	3,301 individuals of European descent	126.409
ebi-a-GCST90012073	Cathepsin L1	32	21,758 individuals of European descent	43.212
prot-a-728	Cathepsin L2	11	3,301 individuals of European descent	24.669
prot-a-726	Cathepsin O	13	3,301 individuals of European descent	23.267
prot-a-727	Cathepsin S	24	3,301 individuals of European descent	39.691
prot-a-729	Cathepsin Z	13	3,301 individuals of European descent	53.426
ebi-a-GCST90038684	Ulcerative colitis	46	2,515 cases and 482,083 controls	32.190
ukb-a-552	Crohn’s disease	25	732 cases and 336,467 controls	26.597

Abbreviation: SNPs, single-nucleotide polymorphisms; IEU, Integrative Epidemiology Unit; GWAS, Genome-wide association studies

### 2.3 Selection of IVs

To satisfy the basic assumption of MR that IVs must be closely related to the exposure factors, SNPs that were significantly associated with various cathepsins (µg/L), including cathepsin B, D, E, F, G, H, L1, L2, O, S, and Z, at the genome-wide level (*p* < 5 × 10^−6^, r2 <0.001, genetic distance = 10,000 kb) were screened. The same criteria were applied for the reverse Mendelian randomization analysis related to ulcerative colitis and Crohn’s disease. Subsequently, the strength of each IV was calculated by the following formula: F = R^2^(N−2)/1−R^2^, in which R^2^ represents the proportion of variability in the cathepsin explained by each IV, and N is the sample size of the GWAS for the SNP–cathepsin association. F-statistics greater than 10 were generally considered to have a strong association (Staiger and Stock, 1997; Bowden et al., 2016a). SNPs in the MR analysis can be found in Supplementary Figure S1 and Supplementary Tables S1, S2.

### 2.4 Statistical analysis

First, the exposure and outcome data sets were harmonized, which ensured the reference allele (EA) remained consistently associated with the same allele across all analyses. Different MR methods (MR-Egger (Bowden et al., 2015), weighted median (Bowden et al., 2016b), IVW (Burgess et al., 2013), simple mode, and weighted mode (Hartwig et al., 2017)) were employed for the two-sample MR analysis to investigate the causal relationship between cathepsins and IBD separately. The heterogeneity of the IVW model was evaluated by Cochran’s Q test, and a Q test *p*-value <0.05 indicated significant heterogeneity. Outliers identified and addressed by the MR-PRESSO method were removed immediately once detected. Then, the MR analyses were performed again. The MR-Egger method, which allows for the existence of non-zero intercepts, was employed to detect directional pleiotropy (Verbanck et al., 2018). Additionally, a leave-one-out analysis was performed to ascertain whether the removal of individual SNPs significantly impacted the results. All statistical analyses were performed using R software (version 4.3.1) with the two-sample MR package (Yavorska and Burgess, 2017).

## 3 Results

### 3.1 Defining the causal relationship between various cathepsins and UC

**FIGURE 2:**
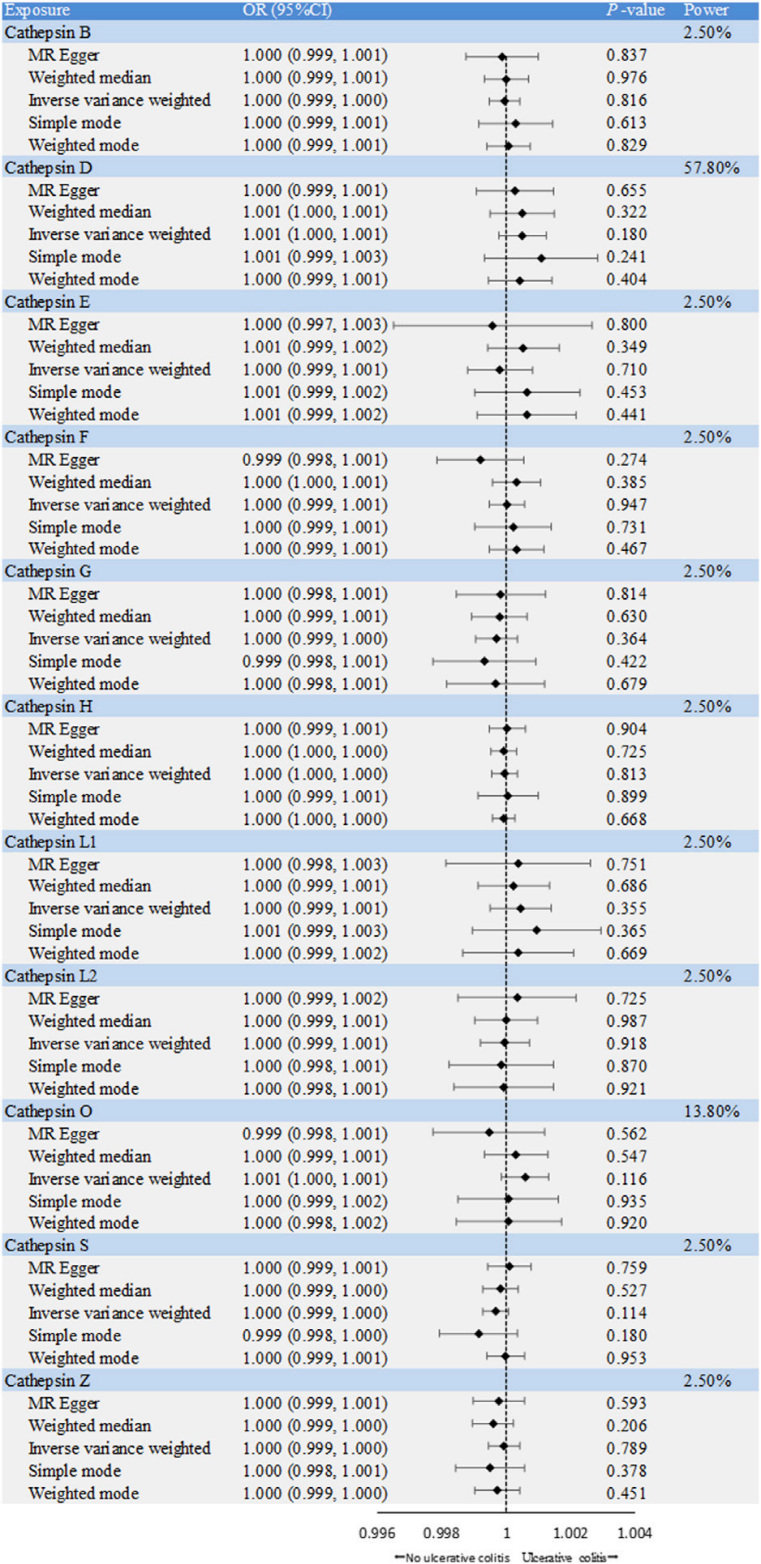
Forest plot of causal effects of cathepsin single-nucleotide polymorphisms (SNPs) on ulcerative colitis. We conducted inverse-variance weighted analyses, among others, to evaluate the causal relationship between cathepsins and ulcerative colitis. (error bars represent 95% confidence intervals).

**FIGURE 3:**
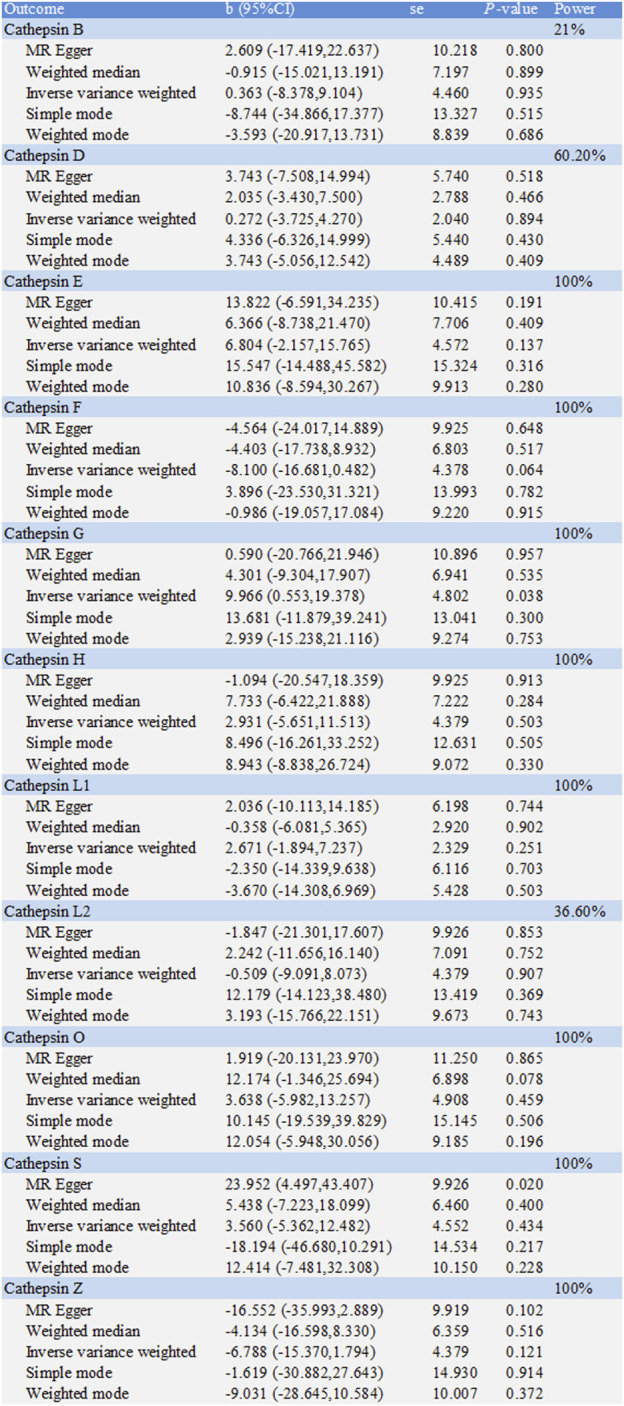
Two-sample Mendelian randomization study based on the MR method estimates the causal effects of ulcerative colitis on cathepsins.

**FIGURE 4:**
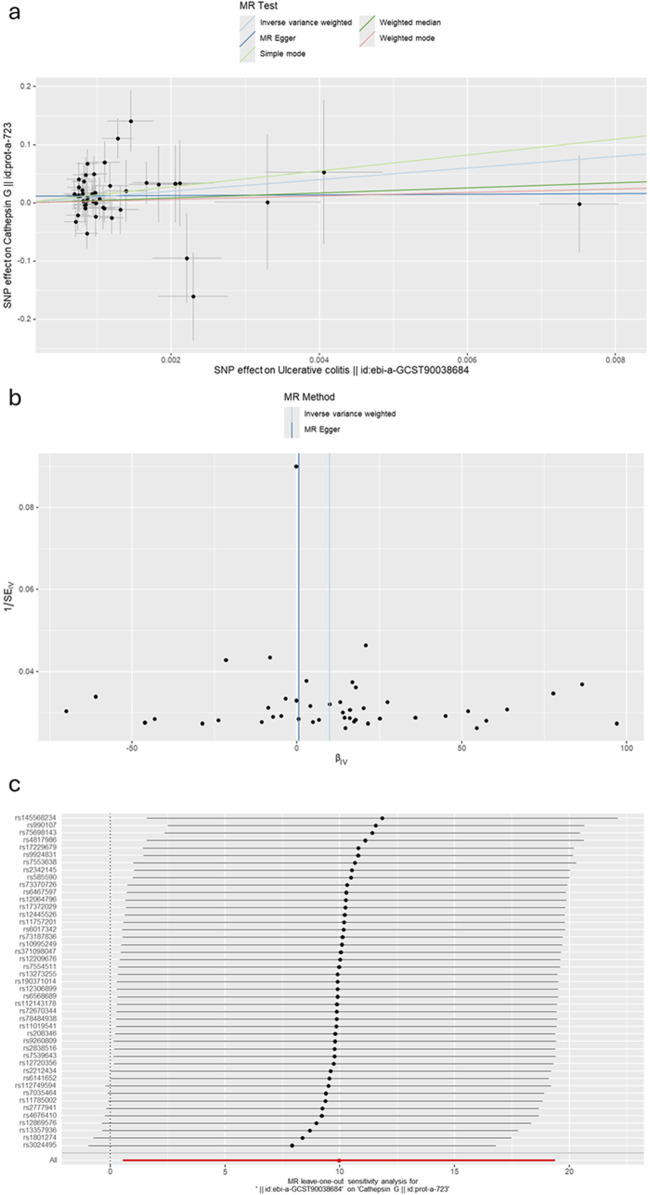
**(A)** A scatter plot represents genetic IV association between ulcerative colitis and cathepsin G. **(B)** A funnel plot of SNPs associated with ulcerative colitis and cathepsin G. **(C)** A leave-one-out analysis of the estimations for ulcerative colitis and cathepsin G.

The causal associations between eleven types of cathepsins (cathepsin B, E, F, G, H, L2, O, S, and Z) and IBD were analyzed. The forward MR analysis did not reveal any causal associations between eleven types of cathepsins and the risk of IBD (Figure 2). We conducted reverse MR analyses to explore the possibility of reverse causality. The reverse MR analysis provided evidence that UC elevated cathepsin G levels (IVW: *p* = 0.038, b = 9.966) (Figures 3, 4), and the *p*-values of the Cochran’s Q test, the MR-PRESSO global test, and the MR-Egger intercept showing no signs of heterogeneity and directional pleiotropy (0.167, 0.168, and 0.343, respectively). The statistical power of this analysis reached 100%. The effect estimate from MR-Egger is 0.590, while the IVW estimate is 9.966. Although the *p*-value for the MR-Egger intercept test is 0.343, we note that the b-value from MR-Egger (0.590) is considerably smaller than its standard error (9.966), which may suggest potential pleiotropy. No evidence supported a causal association between UC and other types of cathepsins (Figure 3).

### 3.2 Defining the causal relationship between various cathepsins and CD

**FIGURE 5:**
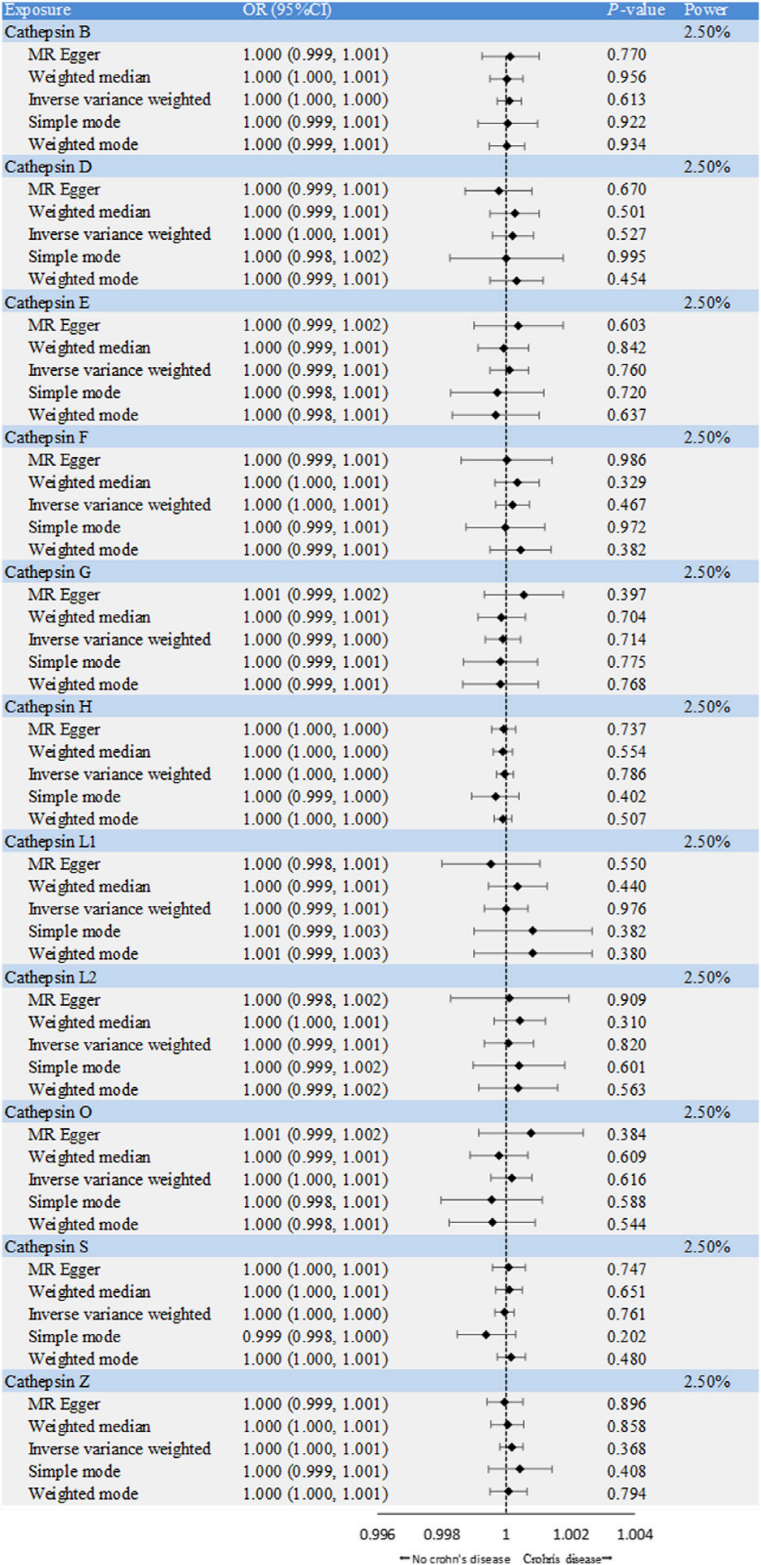
Forest plot of causal effects of cathepsin single-nucleotide polymorphisms (SNPs) on Crohn’s disease. We conducted inverse-variance weighted analyses to evaluate the causal relationship between cathepsins and Crohn’s disease. (error bars represent 95% confidence intervals).

**FIGURE 6:**
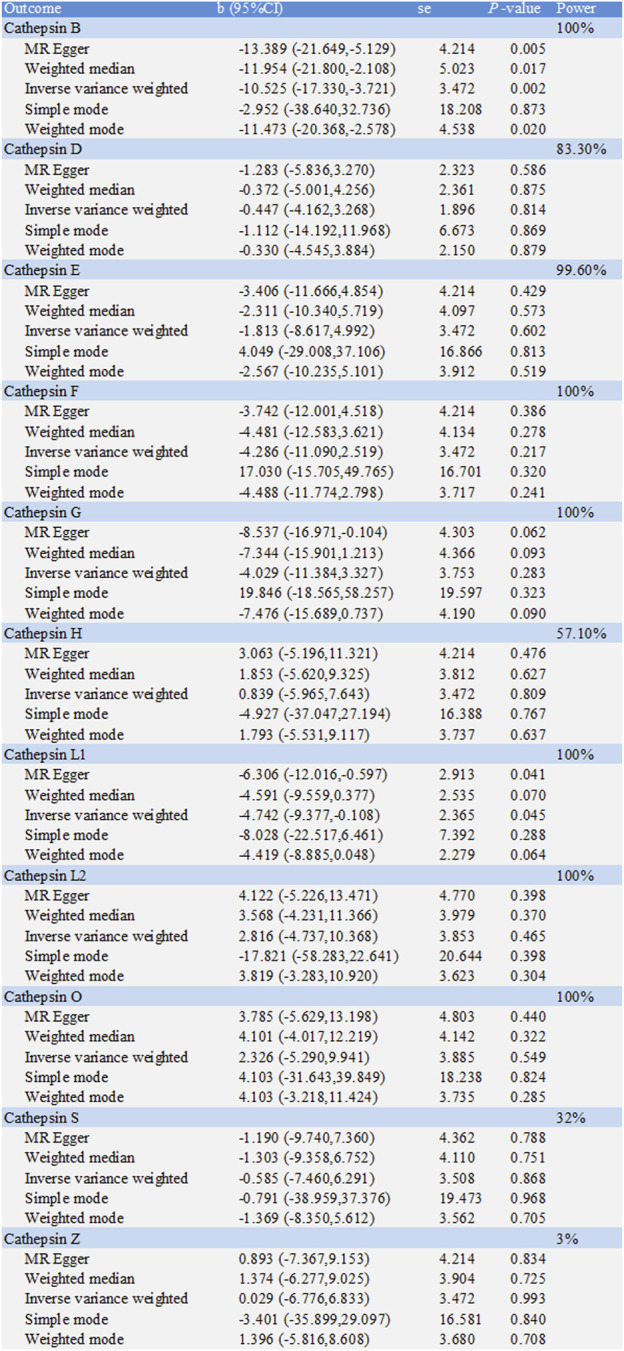
Two-sample Mendelian randomization study based on the MR method estimates the causal effects of Crohn’s disease on cathepsins.

**FIGURE 7:**
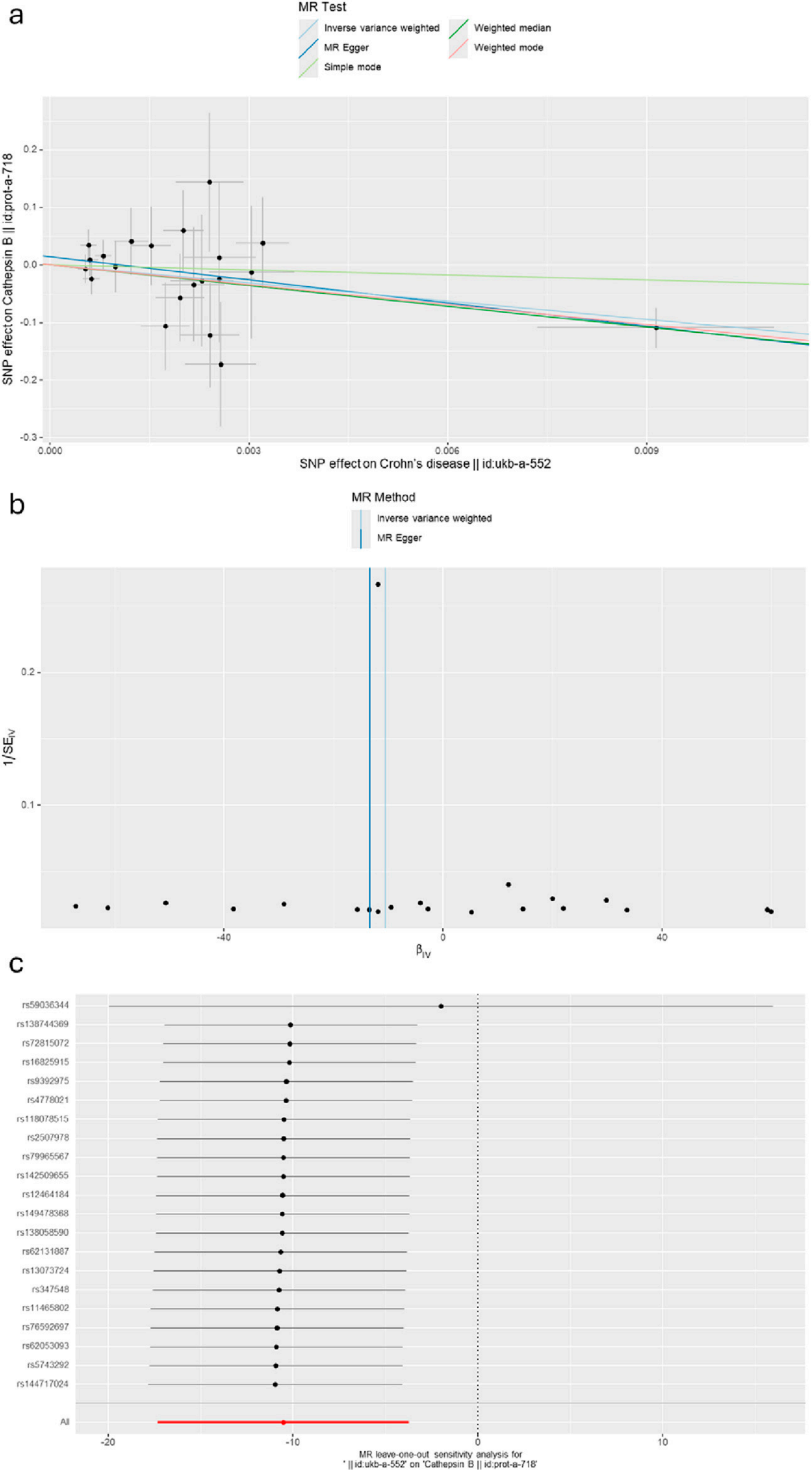
**(A)** A scatter plot represents genetic IV association between Crohn’s disease and cathepsin B. **(B)** A funnel plot of SNPs associated with Crohn’s disease and cathepsin B. **(C)** A leave-one-out analysis of the estimations for Crohn’s disease and cathepsin B.

**FIGURE 8:**
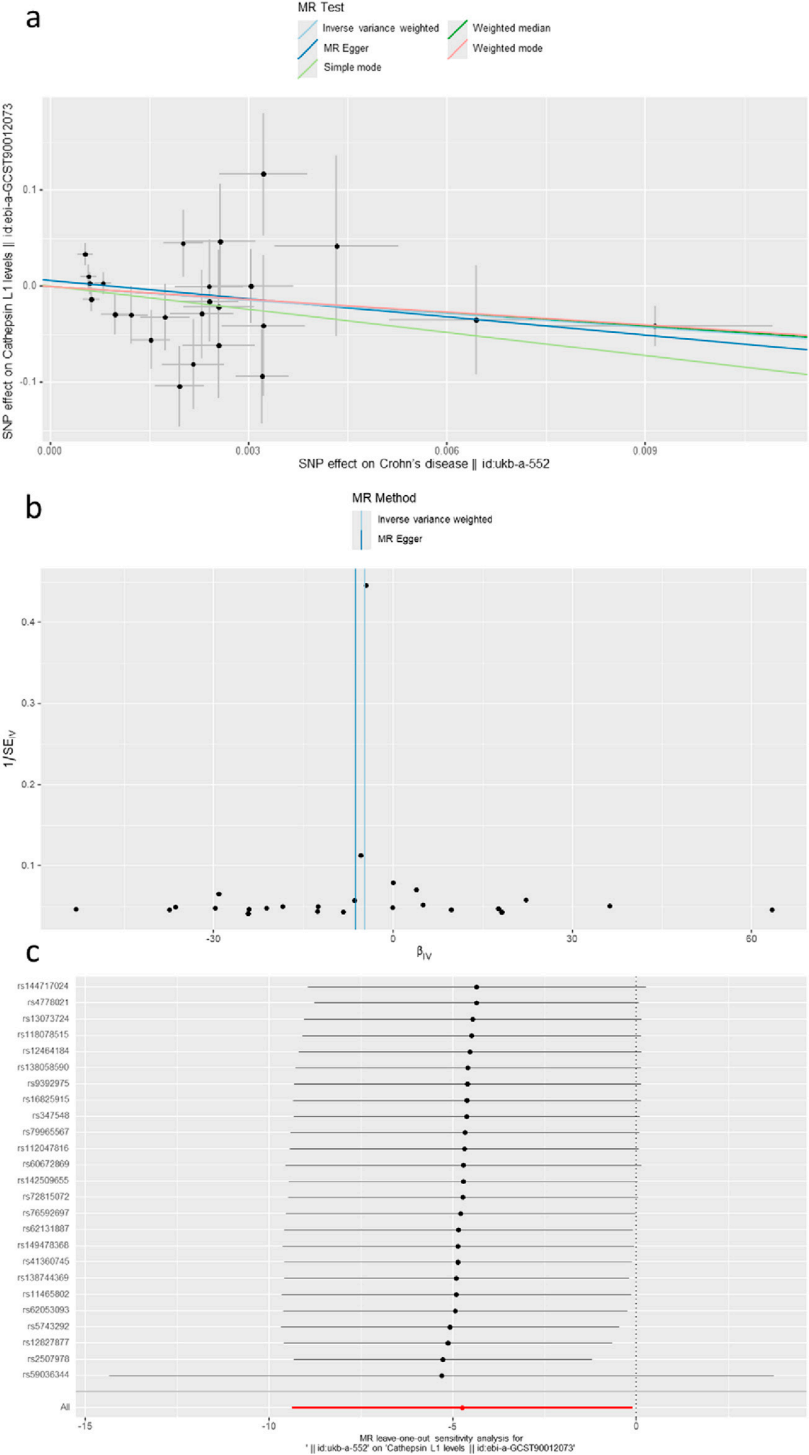
**(A)** A scatter plot represents the genetic IV association between Crohn’s disease and cathepsin L1. **(B)** A funnel plot of SNPs associated with Crohn’s disease and cathepsin L1. **(C)** A leave-one-out analysis of the estimations for Crohn’s disease and cathepsin L1.

The causal associations between eleven types of cathepsins (cathepsin B, D, E, F, G, H, L1, L2, O, S, and Z) and CD were analyzed. The forward MR analysis did not reveal any causal associations between eleven types of cathepsins and CD risk (Figure 5). We conducted reverse MR analyses to explore the possibility of reverse causality. The reverse MR analysis provided evidence that CD decreased the level of cathepsin B (cathepsin B: IVW: *p* = 0.002, b = −10.525) (Figures 6, 7). The *p*-values of Cochran’s Q test, the MR-PRESSO global test, and the MR-Egger intercept showed no signs of heterogeneity, outliers, or directional pleiotropy (0.828, 0.709, and 0.245, respectively). The reverse MR analysis provided evidence that CD decreased the level of cathepsin L1 (IVW: *p* = 0.045, b = −4.742) (Figure 8), and the *p*-value of the Cochran’s Q test was 0.047, suggesting the presence of slight but statistically significant heterogeneity among the genetic instruments deployed. The *p*-values of the MR-PRESSO global test and the MR-Egger intercept showed no signs of outliers or directional pleiotropy (0.147 and 0.365, respectively). The statistical power of these analyses reached 100%. No evidence supported a causal association between CD and other types of cathepsins (Figure 6).

Overall, in primary MR analyses, IBD was statistically associated with cathepsin B and cathepsin G, suggesting the development of IBD might affect the level of these cathepsins.

## 4 Discussion

IBD is a prevalent chronic immune-mediated inflammatory disorder of the digestive tract that, if left untreated, can lead to prolonged bowel damage. Recent observational analyses have suggested that intestinal pathophysiological conditions can disrupt proteolytic homeostasis, resulting in inappropriate substrate degradation and abnormal accumulation (Sekula et al., 2016). Among the key players in these processes, cathepsins have garnered significant attention from researchers. In this study, we conducted a systematic analysis of the causal relationship between eleven distinct cathepsins and IBD using genetic instruments. Our findings revealed that cathepsin levels do not significantly influence the onset or progression of IBD. Reverse Mendelian randomization analyses suggested that ulcerative colitis (UC) might lead to elevated cathepsin G levels, while Crohn’s disease (CD) may cause a decrease in cathepsin B and L1 levels.

Previous observational research partially corroborates our findings regarding the association between UC and cathepsin G. Marta Dabek et al. demonstrated through RT-PCR and Western blotting that Cat-G is overexpressed in UC patients, with higher mRNA expression in biopsies than healthy subjects (0.44 ± 0.08 vs 0.24 ± 0.03, *p* < 0.05) (Dabek et al., 2009). Additionally, Kuwana’s study showed that anti-neutrophil cytoplasmic antibodies targeting Cat-G are present in the sera of more than half of UC patients (Kuwana et al., 2000). These findings suggest that Cat-G may play a crucial role in UC pathogenesis. However, due to the larger uncertainty in the MR-Egger estimate, we cannot entirely rule out the presence of pleiotropy. Thus, further investigation is necessary to confirm the causal relationship between UC and cathepsin G.

Interestingly, our study revealed that CD occurrence correlates with lower levels of cathepsin B and cathepsin L1, which contradicts previous observational research and clinical studies. Menzel et al. confirmed the upregulation of cathepsin B and cathepsin L in areas of tissue damage and mucosal ulceration in IBD patients using immunohistochemistry and gene expression analysis (Menzel et al., 2006). Moreover, elevated cathepsin B levels have been observed in various pathological conditions, including inflammation, infection, and cancer. Benjamin Bian et al. reported increased mRNA and activated levels of cathepsin B in human adenomas and colorectal cancers (CRCs) at all stages (Bian et al., 2016). Zhenhu Zhang’s study demonstrated significantly higher expression levels of CTSL in ESCC tissues than adjacent non-cancerous tissues (Zhang et al., 2024); Shengnan Zhao’s research revealed increased cathepsin B levels in colonic tissue and exacerbated dextran sodium sulfate (DSS)-induced colitis (Zhao et al., 2016). The discrepancy between our findings and previous observational studies could be attributed to bias from reverse causality or residual confounding in the latter. These divergent results underscore the need for further research to elucidate the complex relationship between cathepsins and IBD.

This study exhibits several notable strengths and limitations. First, MR leverages genetic variants to estimate the causal effects of circulating proteins on IBD, effectively overcoming the bias caused by reverse causality and confounding. Second, we performed sensitivity and pleiotropic analyses to ensure the accuracy of MR analysis. We used exposures and outcomes in European populations from different countries to minimize the potential for population stratification bias. Despite these insights, several limitations warrant consideration, such as (1) the potential overlap of participants between the exposure and outcome GWAS in the two-sample MR analyses could not be ascertained in this study; (2) the presence of pleiotropy, where instrumental SNPs may influence multiple traits, could not be entirely ruled out; however, no evidence of pleiotropy was observed in the MR analyses conducted in any of the above-mentioned MR approaches; (3) given that our study cohort comprised exclusively of individuals of European ancestry, caution should be exercised when generalizing these findings to broader populations.

In conclusion, this study’s primary genetic evidence reveals a nuanced relationship between cathepsins and IBD. Cathepsins do not appear to have a direct impact on IBD. While the occurrence of UC may be associated with elevated levels of cathepsin G, CD was linked to lower levels of cathepsins B and L1. Future research should aim to definitively establish and validate these causal relationships. These insights may prove valuable in identifying biochemical markers for the prediction, screening, early diagnosis, and prognosis of IBD.

## Data Availability

The original contributions presented in the study are included in the article/Supplementary Material; further inquiries can be directed to the corresponding author.
