# Supplementary material for: Bidirectional two-sample Mendelian randomization analysis investigates causal associations between cathepsins and inflammatory bowel disease
Source: Front Genet. 2024 Sep 18;15:1436407. doi: 10.3389/fgene.2024.1436407 (PMC11445167; doi:10.3389/fgene.2024.1436407)
Supplement: Supplementary file 3 [file Table1.DOCX]

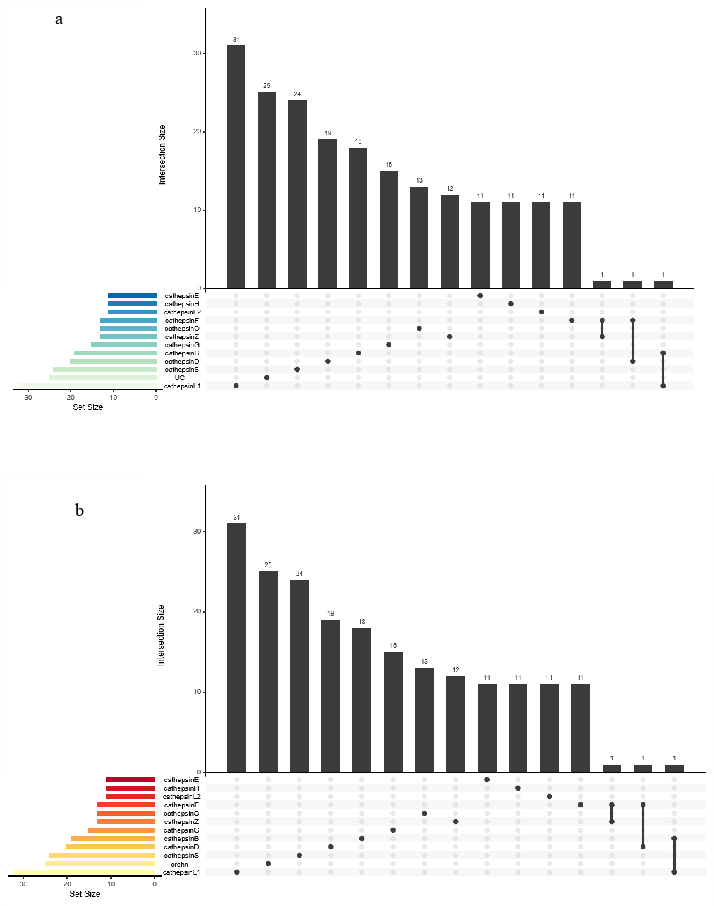


Figure S1. UpSet plot illustrating the intersection of various cathepsin types and ulcerative colitis (a). UpSet plot illustrating the intersection of various cathepsin types and Crohn's disease (b). The upper panel displays a bar chart representing the size of intersections between different sets, with the y-axis indicating the intersection size. The lower panel shows a matrix where rows represent individual sets, and columns correspond to specific intersections. Filled circles in the matrix indicate which sets contribute to each intersection.
