# Supplementary material for: Bidirectional two-sample Mendelian randomization analysis investigates causal associations between cathepsins and inflammatory bowel disease
Source: Front Genet. 2024 Sep 18;15:1436407. doi: 10.3389/fgene.2024.1436407 (PMC11445167; doi:10.3389/fgene.2024.1436407)
Supplement: Supplementary file 4 [file DataSheet2.DOCX]

##Bi-directional Mendelian Randomization Analysis of Cathepsins and Ulcerative Colitis

library(TwoSampleMR)

library(ieugwasr)

library(MRInstruments)

library(MRlap)

library(dplyr)

library(xlsx)

library(tidyverse)

#--------------------------------- exposure --------------------------------

cathepsinB <- extract_instruments(outcomes="prot-a-718",p1 = 5e-06)

cathepsinD <- extract_instruments(outcomes="ebi-a-GCST90012053",p1 = 5e-06)

cathepsinE <- extract_instruments(outcomes="prot-a-720",p1 = 5e-06)

cathepsinF <- extract_instruments(outcomes="prot-a-722",p1 = 5e-06)

cathepsinG <- extract_instruments(outcomes="prot-a-723",p1 = 5e-06)

cathepsinH <- extract_instruments(outcomes="prot-a-725",p1 = 5e-06)

cathepsinL1 <- extract_instruments(outcomes="ebi-a-GCST90012073",p1 = 5e-06)

cathepsinL2 <- extract_instruments(outcomes="prot-a-728",p1 = 5e-06)

cathepsinO <- extract_instruments(outcomes="prot-a-726",p1 = 5e-06)

cathepsinS <- extract_instruments(outcomes="prot-a-727",p1 = 5e-06)

cathepsinZ <- extract_instruments(outcomes="prot-a-729",p1 = 5e-06)

#------------------------------- UC outcome-------------------------------

UC_cathepsinB <- extract_outcome_data(snps = cathepsinB$SNP, outcomes = "ebi-a-GCST90038684")

UC_cathepsinE <- extract_outcome_data(snps = cathepsinE$SNP, outcomes = "ebi-a-GCST90038684")

UC_cathepsinD <- extract_outcome_data(snps = cathepsinD$SNP, outcomes = "ebi-a-GCST90038684")

UC_cathepsinF <- extract_outcome_data(snps = cathepsinF$SNP, outcomes = "ebi-a-GCST90038684")

UC_cathepsinG <- extract_outcome_data(snps = cathepsinG$SNP, outcomes = "ebi-a-GCST90038684")

UC_cathepsinH <- extract_outcome_data(snps = cathepsinH$SNP, outcomes = "ebi-a-GCST90038684")

UC_cathepsinL1 <- extract_outcome_data(snps = cathepsinL1$SNP, outcomes = "ebi-a-GCST90038684")

UC_cathepsinL2 <- extract_outcome_data(snps = cathepsinL2$SNP, outcomes = "ebi-a-GCST90038684")

UC_cathepsinO <- extract_outcome_data(snps = cathepsinO$SNP, outcomes = "ebi-a-GCST90038684")

UC_cathepsinS <- extract_outcome_data(snps = cathepsinS$SNP, outcomes = "ebi-a-GCST90038684")

UC_cathepsinZ <- extract_outcome_data(snps = cathepsinZ$SNP, outcomes = "ebi-a-GCST90038684")

#------------------------------ Harmonization ------------------------------

cathepsinB_UC <- harmonise_data(exposure_dat = cathepsinB,outcome_dat = UC_cathepsinB)

cathepsinD_UC <- harmonise_data(exposure_dat = cathepsinD,outcome_dat = UC_cathepsinD)

cathepsinE_UC <- harmonise_data(exposure_dat = cathepsinE,outcome_dat = UC_cathepsinE)

cathepsinF_UC <- harmonise_data(exposure_dat = cathepsinF,outcome_dat = UC_cathepsinF)

cathepsinG_UC <- harmonise_data(exposure_dat = cathepsinG,outcome_dat = UC_cathepsinG)

cathepsinH_UC <- harmonise_data(exposure_dat = cathepsinH,outcome_dat = UC_cathepsinH)

cathepsinL1_UC <- harmonise_data(exposure_dat = cathepsinL1,outcome_dat = UC_cathepsinL1)

cathepsinL2_UC <- harmonise_data(exposure_dat = cathepsinL2,outcome_dat = UC_cathepsinL2)

cathepsinO_UC <- harmonise_data(exposure_dat = cathepsinO,outcome_dat = UC_cathepsinO)

cathepsinS_UC <- harmonise_data(exposure_dat = cathepsinS,outcome_dat = UC_cathepsinS)

cathepsinZ_UC <- harmonise_data(exposure_dat = cathepsinZ,outcome_dat = UC_cathepsinZ)

#--------------------------------- MR ---------------------------------

generate_odds_ratios(mr(cathepsinB_UC))

generate_odds_ratios(mr(cathepsinD_UC))

generate_odds_ratios(mr(cathepsinE_UC))

generate_odds_ratios(mr(cathepsinF_UC))

generate_odds_ratios(mr(cathepsinG_UC))

generate_odds_ratios(mr(cathepsinH_UC))

generate_odds_ratios(mr(cathepsinL1_UC))

generate_odds_ratios(mr(cathepsinL2_UC))

generate_odds_ratios(mr(cathepsinO_UC))

generate_odds_ratios(mr(cathepsinS_UC))

generate_odds_ratios(mr(cathepsinZ_UC))

#-------------------reverse Mendelian randomization analyses------------------

#-------------------------------- exposure ---------------------------------

UC <- extract_instruments(outcomes="ebi-a-GCST90038684",p1 = 5e-06)

#-------------------------------- outcome ---------------------------------

cathB_UC <- extract_outcome_data(snps = UC$SNP, outcomes = "prot-a-718")

cathD_UC <- extract_outcome_data(snps = UC$SNP, outcomes = "ebi-a-GCST90012053")

cathE_UC <- extract_outcome_data(snps = UC$SNP, outcomes = "prot-a-720")

cathF_UC <- extract_outcome_data(snps = UC$SNP, outcomes = "prot-a-722")

cathG_UC <- extract_outcome_data(snps = UC$SNP, outcomes = "prot-a-723")

cathH_UC <- extract_outcome_data(snps = UC$SNP, outcomes = "prot-a-725")

cathL1_UC <- extract_outcome_data(snps = UC$SNP, outcomes = "ebi-a-GCST90012073")

cathL2_UC <- extract_outcome_data(snps = UC$SNP, outcomes = "prot-a-728")

cathO_UC <- extract_outcome_data(snps = UC$SNP, outcomes = "prot-a-726")

cathS_UC <- extract_outcome_data(snps = UC$SNP, outcomes = "prot-a-727")

cathZ_UC <- extract_outcome_data(snps = UC$SNP, outcomes = "prot-a-729")

#------------------------------ Harmonization ------------------------------

UC_cathB <- harmonise_data(exposure_dat = UC,outcome_dat = cathB_UC)

UC_cathD <- harmonise_data(exposure_dat = UC,outcome_dat = cathD_UC)

UC_cathE <- harmonise_data(exposure_dat = UC,outcome_dat = cathE_UC)

UC_cathF <- harmonise_data(exposure_dat = UC,outcome_dat = cathF_UC)

UC_cathG <- harmonise_data(exposure_dat = UC,outcome_dat = cathG_UC)

UC_cathH <- harmonise_data(exposure_dat = UC,outcome_dat = cathH_UC)

UC_cathL1 <- harmonise_data(exposure_dat = UC,outcome_dat = cathL1_UC)

UC_cathL2 <- harmonise_data(exposure_dat = UC,outcome_dat = cathL2_UC)

UC_cathO <- harmonise_data(exposure_dat = UC,outcome_dat = cathO_UC)

UC_cathS <- harmonise_data(exposure_dat = UC,outcome_dat = cathS_UC)

UC_cathZ <- harmonise_data(exposure_dat = UC,outcome_dat = cathZ_UC)

#--------------------------------- MR ---------------------------------

generate_odds_ratios(mr(UC_cathB))

generate_odds_ratios(mr(UC_cathD))

generate_odds_ratios(mr(UC_cathE))

generate_odds_ratios(mr(UC_cathF))

generate_odds_ratios(mr(UC_cathG))

generate_odds_ratios(mr(UC_cathH))

generate_odds_ratios(mr(UC_cathL1))

generate_odds_ratios(mr(UC_cathL2))

generate_odds_ratios(mr(UC_cathO))

generate_odds_ratios(mr(UC_cathS))

generate_odds_ratios(mr(UC_cathZ))

#-----------------------------------figure--------------------------------

mr_scatter_plot(mr_results=mr(UC_cathG),UC_cathG)

mr_heterogeneity(UC_cathG)

run_mr_presso(UC_cathG,NbDistribution = 3000)

mr_funnel_plot(singlesnp_results = mr_singlesnp(UC_cathG))

mr_pleiotropy_test(UC_cathG)

mr_leaveoneout_plot(mr_leaveoneout(UC_cathG))

##Bi-directional Mendelian Randomization Analysis of Cathepsins and Crohn’s Disease

library(TwoSampleMR)

library(ieugwasr)

library(MRInstruments)

library(MRlap)

library(dplyr)

library(xlsx)

library(tidyverse)

#--------------------------------- exposure --------------------------------

cathepsinB <- extract_instruments(outcomes="prot-a-718",p1 = 5e-06)

cathepsinD <- extract_instruments(outcomes="ebi-a-GCST90012053",p1 = 5e-06)

cathepsinE <- extract_instruments(outcomes="prot-a-720",p1 = 5e-06)

cathepsinF <- extract_instruments(outcomes="prot-a-722",p1 = 5e-06)

cathepsinG <- extract_instruments(outcomes="prot-a-723",p1 = 5e-06)

cathepsinH <- extract_instruments(outcomes="prot-a-725",p1 = 5e-06)

cathepsinL1 <- extract_instruments(outcomes="ebi-a-GCST90012073",p1 = 5e-06)

cathepsinL2 <- extract_instruments(outcomes="prot-a-728",p1 = 5e-06)

cathepsinO <- extract_instruments(outcomes="prot-a-726",p1 = 5e-06)

cathepsinS <- extract_instruments(outcomes="prot-a-727",p1 = 5e-06)

cathepsinZ <- extract_instruments(outcomes="prot-a-729",p1 = 5e-06)

#------------------------ crohn’s disease outcome----------------------------

crohn_cathepsinB <- extract_outcome_data(snps = cathepsinB$SNP, outcomes = "ukb-a-552")

crohn_cathepsinE <- extract_outcome_data(snps = cathepsinE$SNP, outcomes = "ukb-a-552")

crohn_cathepsinD <- extract_outcome_data(snps = cathepsinD$SNP, outcomes = "ukb-a-552")

crohn_cathepsinF <- extract_outcome_data(snps = cathepsinF$SNP, outcomes = "ukb-a-552")

crohn_cathepsinG <- extract_outcome_data(snps = cathepsinG$SNP, outcomes = "ukb-a-552")

crohn_cathepsinH <- extract_outcome_data(snps = cathepsinH$SNP, outcomes = "ukb-a-552")

crohn_cathepsinL1 <- extract_outcome_data(snps = cathepsinL1$SNP, outcomes = "ukb-a-552")

crohn_cathepsinL2 <- extract_outcome_data(snps = cathepsinL2$SNP, outcomes = "ukb-a-552")

crohn_cathepsinO <- extract_outcome_data(snps = cathepsinO$SNP, outcomes = "ukb-a-552")

crohn_cathepsinS <- extract_outcome_data(snps = cathepsinS$SNP, outcomes = "ukb-a-552")

crohn_cathepsinZ <- extract_outcome_data(snps = cathepsinZ$SNP, outcomes = "ukb-a-552")

#------------------------------ Harmonization ------------------------------

cathepsinB_crohn <- harmonise_data(exposure_dat = cathepsinB,outcome_dat = crohn_cathepsinB)

cathepsinD_crohn <- harmonise_data(exposure_dat = cathepsinD,outcome_dat = crohn_cathepsinD)

cathepsinE_crohn <- harmonise_data(exposure_dat = cathepsinE,outcome_dat = crohn_cathepsinE)

cathepsinF_crohn <- harmonise_data(exposure_dat = cathepsinF,outcome_dat = crohn_cathepsinF)

cathepsinG_crohn <- harmonise_data(exposure_dat = cathepsinG,outcome_dat = crohn_cathepsinG)

cathepsinH_crohn <- harmonise_data(exposure_dat = cathepsinH,outcome_dat = crohn_cathepsinH)

cathepsinL1_crohn <- harmonise_data(exposure_dat = cathepsinL1,outcome_dat = crohn_cathepsinL1)

cathepsinL2_crohn <- harmonise_data(exposure_dat = cathepsinL2,outcome_dat = crohn_cathepsinL2)

cathepsinO_crohn <- harmonise_data(exposure_dat = cathepsinO,outcome_dat = crohn_cathepsinO)

cathepsinS_crohn <- harmonise_data(exposure_dat = cathepsinS,outcome_dat = crohn_cathepsinS)

cathepsinZ_crohn <- harmonise_data(exposure_dat = cathepsinZ,outcome_dat = crohn_cathepsinZ)

#--------------------------------- MR ---------------------------------

generate_odds_ratios(mr(cathepsinB_crohn))

generate_odds_ratios(mr(cathepsinD_crohn))

generate_odds_ratios(mr(cathepsinE_crohn))

generate_odds_ratios(mr(cathepsinF_crohn))

generate_odds_ratios(mr(cathepsinG_crohn))

generate_odds_ratios(mr(cathepsinH_crohn))

generate_odds_ratios(mr(cathepsinL1_crohn))

generate_odds_ratios(mr(cathepsinL2_crohn))

generate_odds_ratios(mr(cathepsinO_crohn))

generate_odds_ratios(mr(cathepsinS_crohn))

generate_odds_ratios(mr(cathepsinZ_crohn))

#-------------------reverse Mendelian randomization analyses------------------

#-------------------------------- exposure ---------------------------------

crohn <- extract_instruments(outcomes="ukb-a-552",p1 = 5e-06)

#-------------------------------- outcome ---------------------------------

cathB_crohn <- extract_outcome_data(snps = crohn$SNP, outcomes = "prot-a-718")

cathD_crohn <- extract_outcome_data(snps = crohn$SNP, outcomes = "ebi-a-GCST90012053")

cathE_crohn <- extract_outcome_data(snps = crohn$SNP, outcomes = "prot-a-720")

cathF_crohn <- extract_outcome_data(snps = crohn$SNP, outcomes = "prot-a-722")

cathG_crohn <- extract_outcome_data(snps = crohn$SNP, outcomes = "prot-a-723")

cathH_crohn <- extract_outcome_data(snps = crohn$SNP, outcomes = "prot-a-725")

cathL1_crohn <- extract_outcome_data(snps = crohn$SNP, outcomes = "ebi-a-GCST90012073")

cathL2_crohn <- extract_outcome_data(snps = crohn$SNP, outcomes = "prot-a-728")

cathO_crohn <- extract_outcome_data(snps = crohn$SNP, outcomes = "prot-a-726")

cathS_crohn <- extract_outcome_data(snps = crohn$SNP, outcomes = "prot-a-727")

cathZ_crohn <- extract_outcome_data(snps = crohn$SNP, outcomes = "prot-a-729")

#------------------------------ Harmonization ------------------------------

crohn_cathB <- harmonise_data(exposure_dat = crohn,outcome_dat = cathB_crohn)

crohn_cathD <- harmonise_data(exposure_dat = crohn,outcome_dat = cathD_crohn)

crohn_cathE <- harmonise_data(exposure_dat = crohn,outcome_dat = cathE_crohn)

crohn_cathF <- harmonise_data(exposure_dat = crohn,outcome_dat = cathF_crohn)

crohn_cathG <- harmonise_data(exposure_dat = crohn,outcome_dat = cathG_crohn)

crohn_cathH <- harmonise_data(exposure_dat = crohn,outcome_dat = cathH_crohn)

crohn_cathL1 <- harmonise_data(exposure_dat = crohn,outcome_dat = cathL1_crohn)

crohn_cathL2 <- harmonise_data(exposure_dat = crohn,outcome_dat = cathL2_crohn)

crohn_cathO <- harmonise_data(exposure_dat = crohn,outcome_dat = cathO_crohn)

crohn_cathS <- harmonise_data(exposure_dat = crohn,outcome_dat = cathS_crohn)

crohn_cathZ <- harmonise_data(exposure_dat = crohn,outcome_dat = cathZ_crohn)

#--------------------------------- MR ---------------------------------

generate_odds_ratios(mr(crohn_cathB))

generate_odds_ratios(mr(crohn_cathD))

generate_odds_ratios(mr(crohn_cathE))

generate_odds_ratios(mr(crohn_cathF))

generate_odds_ratios(mr(crohn_cathG))

generate_odds_ratios(mr(crohn_cathH))

generate_odds_ratios(mr(crohn_cathL1))

generate_odds_ratios(mr(crohn_cathL2))

generate_odds_ratios(mr(crohn_cathO))

generate_odds_ratios(mr(crohn_cathS))

generate_odds_ratios(mr(crohn_cathZ))

#-----------------------------------figure--------------------------------

mr_scatter_plot(mr_results=mr(crohn_cathB),crohn_cathB)

mr_heterogeneity(crohn_cathB)

run_mr_presso(crohn_cathB,NbDistribution = 3000)

mr_funnel_plot(singlesnp_results = mr_singlesnp(crohn_cathB))

mr_pleiotropy_test(crohn_cathB)

mr_leaveoneout_plot(mr_leaveoneout(crohn_cathB))

mr_scatter_plot(mr_results=mr(crohn_cathL1),crohn_cathL1)

mr_heterogeneity(crohn_cathL1)

run_mr_presso(crohn_cathL1,NbDistribution = 3000)

mr_funnel_plot(singlesnp_results = mr_singlesnp(crohn_cathL1))

mr_pleiotropy_test(crohn_cathL1)

mr_leaveoneout_plot(mr_leaveoneout(crohn_cathL1))
