# Supplementary material for: Bidirectional two-sample Mendelian randomization analysis investigates causal associations between cathepsins and inflammatory bowel disease
Source: Front Genet. 2024 Sep 18;15:1436407. doi: 10.3389/fgene.2024.1436407 (PMC11445167; doi:10.3389/fgene.2024.1436407)
Supplement: Supplementary file 5 [file DataSheet1.DOCX]

**STROBE-MR checklist of recommended items to address in reports of Mendelian randomization studies**^1^ ^2^

| **Item No.** | **Section** | **Checklist item** | **Page No.** | **Relevant text from manuscript** |
| --- | --- | --- | --- | --- |
| 1 | **TITLE and ABSTRACT** | Indicate Mendelian randomization (MR) as the study’s design in the title and/or the abstract if that is a main purpose of the study | 1 | Two-Sample Mendelian Randomization Analysis Investigates Causal Associations Between Cathepsins and Inflammatory Bowel Disease |
|  | **INTRODUCTION** |  |  |  |
| 2 | **Background** | Explain the scientific background and rationale for the reported study. What is the exposure? Is a potential causal relationship between exposure and outcome plausible? Justify why MR is a helpful method to address the study question | 1-2 | However, our comprehension of the association between other members of the cathepsin family and IBD remains limited, warranting further exploration and investigation. Meanwhile, due to the methodological constraints of observational studies, causal relationships between cathepsins and IBD cannot be conclusively established. |
| 3 | **Objectives** | State specific objectives clearly, including pre-specified causal hypotheses (if any). State that MR is a method that, under specific assumptions, intends to estimate causal effects | 1-2 | This study utilized Mendelian randomization analyses to evaluate the causal associations between eleven cathepsins and the risk of the Ulcerative colitis and Crohn’s disease. |
|  | **METHODS** |  |  |  |
| 4 | **Study design and data sources** | Present key elements of the study design early in the article. Consider including a table listing sources of data for all phases of the study. For each data source contributing to the analysis, describe the following: | 3,8 | More information about the exposure and outcome datasets is presented in Table 1. |
|  | a) | Setting: Describe the study design and the underlying population, if possible. Describe the setting, locations, and relevant dates, including periods of recruitment, exposure, follow-up, and data collection, when available. | 8 | Table 1 |
|  | b) | Participants: Give the eligibility criteria, and the sources and methods of selection of participants. Report the sample size, and whether any power or sample size calculations were carried out prior to the main analysis | 8 | Table 1 |
|  | c) | Describe measurement, quality control and selection of genetic variants | 3 | SNPs were screened significantly associated with various cathepsins (µg/L), including cathepsin B, D, E, F, G, H, L1, L2, O, S, and Z, at the genome-wide level (P <5×10-6, r2 <0.001, genetic distance = 10,000 kb). Subsequently, the strength of each IV was calculated by the following formula: F= R2(N−2)/1−R2, in which R^2 represents the proportion of variability in the cathepsin explained by each IV, and N is the sample size of the GWAS for the SNP-cathepsin association. The F-statistic greater than 10 was generally considered to have a strong association. |
|  | d) | For each exposure, outcome, and other relevant variables, describe methods of assessment and diagnostic criteria for diseases | 8 | Table 1 |
|  | e) | Provide details of ethics committee approval and participant informed consent, if relevant | 2 | Since the data employed in this study were based on published studies and public databases, no additional ethical approval from an institutional review board was necessary. |
| 5 | **Assumptions** | Explicitly state the three core IV assumptions for the main analysis (relevance, independence and exclusion restriction) as well assumptions for any additional or sensitivity analysis |  | Figure 1 |
| 6 | **Statistical methods: main analysis** | Describe statistical methods and statistics used |  | MR Egger, Weighted median, Inverse variance weighted (IVW), Simple mode, and Weighted mode  Cochran’s Q test, MR-PRESSO |
|  | a) | Describe how quantitative variables were handled in the analyses (i.e., scale, units, model) |  | NA |
|  | b) | Describe how genetic variants were handled in the analyses and, if applicable, how their weights were selected | 3 | MR Egger, Weighted median, Inverse variance weighted (IVW), Simple mode, and Weighted mode |
|  | c) | Describe the MR estimator (e.g. two-stage least squares, Wald ratio) and related statistics. Detail the included covariates and, in case of two-sample MR, whether the same covariate set was used for adjustment in the two samples | 3 | MR Egger, Weighted median, Inverse variance weighted (IVW), Simple mode, and Weighted mode |
|  | d) | Explain how missing data were addressed |  | NA |
|  | e) | If applicable, indicate how multiple testing was addressed |  | NA |
| 7 | **Assessment of assumptions** | Describe any methods or prior knowledge used to assess the assumptions or justify their validity | 3 | MR Egger, Weighted median, Inverse variance weighted (IVW), Simple mode, and Weighted mode |
| 8 | **Sensitivity analyses and additional analyses** | Describe any sensitivity analyses or additional analyses performed (e.g. comparison of effect estimates from different approaches, independent replication, bias analytic techniques, validation of instruments, simulations) | 3 | Cochran’s Q test, MR-PRESSO |
| 9 | **Software and pre-registration** |  |  |  |
|  | a) | Name statistical software and package(s), including version and settings used | 3 | All statistical analyses were performed using R software (version 4.3.1) with the Two Sample MR package. |
|  | b) | State whether the study protocol and details were pre-registered (as well as when and where) |  | NA |
|  | **RESULTS** |  |  |  |
| 10 | **Descriptive data** |  |  |  |
|  | a) | Report the numbers of individuals at each stage of included studies and reasons for exclusion. Consider use of a flow diagram |  | Figure 1 |
|  | b) | Report summary statistics for phenotypic exposure(s), outcome(s), and other relevant variables (e.g. means, SDs, proportions) |  |  |
|  | c) | If the data sources include meta-analyses of previous studies, provide the assessments of heterogeneity across these studies |  | NA |
|  | d) | For two-sample MR:  i.  Provide justification of the similarity of the genetic variant-exposure associations between the exposure and outcome samples  ii.  Provide information on the number of individuals who overlap between the exposure and outcome studies |  | Influence of genetically determined cathepsin levels on IBD risk  Effect of Genetically Predicted IBD Risk on Cathepsin Levels |
| 11 | **Main results** |  |  |  |
|  | a) | Report the associations between genetic variant and exposure, and between genetic variant and outcome, preferably on an interpretable scale | 3-4 | The reverse MR analysis provided evidence that UC elevated cathepsin G levels (IVW: P = 0.038, b = 9.966) (Table 2, Figure 3, Figure 4 and Figure 5), and the P-values of the Cochran’s Q-test, MR-PRESSO global test and MR-Egger intercept showing no signs of heterogeneity and directional pleiotropy (0.167, 0.168 and 0.343, respectively).  The reverse MR analysis provided evidence that CD decreased cathepsin B levels (IVW: P = 0.935, b = 0.363) (Table 3, Figure 7, Figure 8 and Figure 9), and the P-values of the Cochran’s Q-test, MR-PRESSO global test and MR-Egger intercept showing no signs of heterogeneity, outliers, and directional pleiotropy (0.828, 0.709 and 0.245, respectively). |
|  | b) | Report MR estimates of the relationship between exposure and outcome, and the measures of uncertainty from the MR analysis, on an interpretable scale, such as odds ratio or relative risk per SD difference | 3-4 | The causal associations between nine types of cathepsins (cathepsin B, E, F, G, H, L2, O, S, and Z) and IBD were analyzed. The forward MR analysis did not reveal any causal associations between eleven types of cathepsins and the risk of IBD (Figure 2). To explore the possibility of reverse causality, we conducted reverse MR analyses. The reverse MR analysis provided evidence that UC elevated cathepsin G levels (IVW: P = 0.038, b = 9.966) (Table 2, Figure 3, Figure 4 and Figure 5), and the P-values of the Cochran’s Q-test, MR-PRESSO global test and MR-Egger intercept showing no signs of heterogeneity and directional pleiotropy (0.167, 0.168 and 0.343, respectively). No evidence supported a causal association between UC and other types of cathepsins (Table 2).  The causal associations between eleven types of cathepsins (cathepsin B, D, E, F, G, H, L1, L2, O, S, and Z) and CD were analyzed. The forward MR analysis did not reveal any causal associations between nine types of cathepsins and CD risk (Figure 6). To explore the possibility of reverse causality, we conducted reverse MR analyses. The reverse MR analysis provided evidence that CD decreased cathepsin B levels (IVW: P = 0.935, b = 0.363) (Table 3, Figure 7, Figure 8 and Figure 9), and the P-values of the Cochran’s Q-test, MR-PRESSO global test and MR-Egger intercept showing no signs of heterogeneity, outliers, and directional pleiotropy (0.828, 0.709 and 0.245, respectively). No evidence supported a causal association between CD and other types of cathepsins (Table 3). |
|  | c) | If relevant, consider translating estimates of relative risk into absolute risk for a meaningful time period | 4 |  |
|  | d) | Consider plots to visualize results (e.g. forest plot, scatterplot of associations between genetic variants and outcome versus between genetic variants and exposure) |  | Figure 2-8 |
| 12 | **Assessment of assumptions** |  |  |  |
|  | a) | Report the assessment of the validity of the assumptions | 3-4 |  |
|  | b) | Report any additional statistics (e.g., assessments of heterogeneity across genetic variants, such as *I^2^*, Q statistic or E-value) | 3-4 | The reverse MR analysis provided evidence that UC elevated cathepsin G levels (IVW: P = 0.038, b = 9.966) (Table 2, Figure 3, Figure 4 and Figure 5), and the P-values of the Cochran’s Q-test, MR-PRESSO global test and MR-Egger intercept showing no signs of heterogeneity and directional pleiotropy (0.167, 0.168 and 0.343, respectively).  The reverse MR analysis provided evidence that CD decreased cathepsin B levels (IVW: P = 0.935, b = 0.363) (Table 3, Figure 7, Figure 8 and Figure 9), and the P-values of the Cochran’s Q-test, MR-PRESSO global test and MR-Egger intercept showing no signs of heterogeneity, outliers, and directional pleiotropy (0.828, 0.709 and 0.245, respectively). |
| 13 | **Sensitivity analyses and additional analyses** |  |  |  |
|  | a) | Report any sensitivity analyses to assess the robustness of the main results to violations of the assumptions | 4-5 |  |
|  | b) | Report results from other sensitivity analyses or additional analyses | 4-5 | The causal associations between nine types of cathepsins (cathepsin B, E, F, G, H, L2, O, S, and Z) and IBD were analyzed. The forward MR analysis did not reveal any causal associations between eleven types of cathepsins and the risk of IBD (Figure 2). To explore the possibility of reverse causality, we conducted reverse MR analyses. The reverse MR analysis provided evidence that UC elevated cathepsin G levels (IVW: P = 0.038, b = 9.966) (Table 2, Figure 3, Figure 4 and Figure 5), and the P-values of the Cochran’s Q-test, MR-PRESSO global test and MR-Egger intercept showing no signs of heterogeneity and directional pleiotropy (0.167, 0.168 and 0.343, respectively). No evidence supported a causal association between UC and other types of cathepsins (Table 2). |
|  | c) | Report any assessment of direction of causal relationship (e.g., bidirectional MR) | 4-5 | bidirectional MR |
|  | d) | When relevant, report and compare with estimates from non-MR analyses | 4-5 |  |
|  | e) | Consider additional plots to visualize results (e.g., leave-one-out analyses) |  | Figure 5 and 8 |
|  | **DISCUSSION** |  |  |  |
| 14 | **Key results** | Summarize key results with reference to study objectives | 4-5 | The results provided herein suggested that the occurrence and development of UC were associated with higher levels of cysteine cathepsin G, while the occurrence of CD correlated with lower levels of cysteine cathepsin B. Conversely, reverse Mendelian randomization analyses imply that cathepsin levels do not exert a significant effect on the onset or progression of IBD. |
| 15 | **Limitations** | Discuss limitations of the study, taking into account the validity of the IV assumptions, other sources of potential bias, and imprecision. Discuss both direction and magnitude of any potential bias and any efforts to address them | 5 | Our study exhibits several notable strengths and limitations. Firstly, MR leveraging genetic variants to estimate the causal effects of circulating proteins on IBD, which can effectively overcome the bias caused by reverse causality and confounding. Secondly, we performed sensitivity and pleiotropic analysis to ensure the accuracy of MR analysis. We used European populations from different countries in exposures and outcome to minimize the potential for population stratification bias. Despite these insights, several limitations warrant consideration, such as: (1) potential overlap of participants between the exposure and outcome GWAS in the two-sample MR analyses could not be ascertained in this study; (2) the presence of pleiotropy, where instrumental SNPs may influence multiple traits, could not be entirely ruled out; however, no evidence of pleiotropy was observed in the MR analyses conducted in any of the above-mentioned MR approaches; (2) given that our study cohort comprised exclusively of individuals of European ancestry, caution should be exercised when generalizing these findings to broader populations. |
| 16 | **Interpretation** |  |  |  |
|  | a) | Meaning: Give a cautious overall interpretation of results in the context of their limitations and in comparison with other studies | 4-5 | Therefore, bias from reverse causality or residual confounding in the observational studies could explain the divergent findings compared to MR estimates, the further research is warranted. |
|  | b) | Mechanism: Discuss underlying biological mechanisms that could drive a potential causal relationship between the investigated exposure and the outcome, and whether the gene-environment equivalence assumption is reasonable. Use causal language carefully, clarifying that IV estimates may provide causal effects only under certain assumptions |  |  |
|  | c) | Clinical relevance: Discuss whether the results have clinical or public policy relevance, and to what extent they inform effect sizes of possible interventions | 5 | In conclusion, the primary genetic evidence from this study reveals that cathepsins have no impact on IBD, while the occurrence of UC was associated with the higher levels of cathepsin G, the occurrence of CD was associated with the lower levels of cathepsin B. This insight may aid in identifying biochemical markers for the prediction, screening, early diagnosis, and prognosis of IBD. |
| 17 | **Generalizability** | Discuss the generalizability of the study results (a) to other populations, (b) across other exposure periods/timings, and (c) across other levels of exposure | 5 | Given that our study cohort comprised exclusively of individuals of European ancestry, caution should be exercised when generalizing these findings to broader populations. |
|  | **OTHER INFORMATION** |  |  |  |
| 18 | **Funding** | Describe sources of funding and the role of funders in the present study and, if applicable, sources of funding for the databases and original study or studies on which the present study is based | 5 | Grant information is now as follows: Na Wang is supported by the Talent Introduction and Research Initiation Project from Shanxi Province of China (2023RC48); Xuena Wang is supported by the Talent Introduction and Research Initiation Project from Shanxi Province of China (2023RC44). |
| 19 | **Data and data sharing** | Provide the data used to perform all analyses or report where and how the data can be accessed, and reference these sources in the article. Provide the statistical code needed to reproduce the results in the article, or report whether the code is publicly accessible and if so, where |  | NA |
| 20 | **Conflicts of Interest** | All authors should declare all potential conflicts of interest | 5 | The authors declare that they have no competing interests. |

This checklist is copyrighted by the Equator Network under the Creative Commons Attribution 3.0 Unported (CC BY 3.0) license.

1. Skrivankova VW, Richmond RC, Woolf BAR, Yarmolinsky J, Davies NM, Swanson SA, et al. Strengthening the Reporting of Observational Studies in Epidemiology using Mendelian Randomization (STROBE-MR) Statement. JAMA. 2021;under review.

2. Skrivankova VW, Richmond RC, Woolf BAR, Davies NM, Swanson SA, VanderWeele TJ, et al. Strengthening the Reporting of Observational Studies in Epidemiology using Mendelian Randomisation (STROBE-MR): Explanation and Elaboration. BMJ. 2021;375:n2233.
